# Brazil’s benign breast disease care profile and geospatial analysis

**DOI:** 10.31744/einstein_journal/2025AO1132

**Published:** 2025-03-28

**Authors:** Dayan Sansone, Daniela Farah, Afonso Celso Pinto Nazario, Marcelo Cunio Machado Fonseca

**Affiliations:** 1 Department of Gynecology Universidade Federal de São Paulo São Paulo SP Brazil Department of Gynecology, Universidade Federal de São Paulo, São Paulo, SP, Brazil.; 2 Department of Gynecology Health Technologies Assessment Center Universidade Federal de São Paulo São Paulo SP Brazil Department of Gynecology, Health Technologies Assessment Center, Universidade Federal de São Paulo, São Paulo, SP, Brazil.

**Keywords:** Breast diseases, Breast neoplasms, Incidence, Strategy planning, Hospitalization, Demography, Epidemiology, Public health, Unified Health System, Primary healthcare, Healthcare costs, Health expenditures, Brazil

## Abstract

**Objective:**

To quantitatively and geospatially analyze coverage and displacement for the diagnosis and treatment of benign breast diseases in the Brazilian Unified Health System between 2008 and 2019.

**Methods:**

Datasets from the Brazilian Ministry of Health were used to survey medical care for benign breast diseases in Brazil from 2008 to 2019. These data allowed for the determination of benign breast disease treatments, diagnoses, and patient displacement in each region of the country.

**Results:**

Most outpatient consultations were for diagnostic procedures. Approximately 4 million diagnostic procedures and 360,000 hospitalizations for treatment, of which 97% were surgeries, were conducted in this 12-year period. During this period, medical care for benign breast diseases cost more than USD 65 million. The treatment coverage and displacement rates of the female population differed by country region, with the North and Midwest showing patterns different from those of the other three major regions.

**Conclusion:**

Addressing disparities in healthcare service access, regardless of their source, by increasing the service capacity of existing facilities and ensuring correct resource allocation by the healthcare system is crucial.

## In Brief

We conducted a quantitative and geospatial analysis of coverage and displacement for the diagnosis and treatment of benign breast illnesses in Brazil’s Unified Health System between 2008 and 2019. The results showed that treatment coverage and female displacement differed by region. The North and Midwest have different coverage and displacement rates compared with the other three major regions of the country. These findings demonstrate the importance of addressing gaps in healthcare service access, irrespective of their source, by enhancing the service capacity of existing institutions and ensuring that the healthcare system appropriately allocates resources.

## Highlights

■ Most outpatient consultations comprised diagnostic procedures.

■ In the past 12 years, medical care for benign breast diseases has cost >USD 65 million.

■ Women from North and Midwest Brazil had higher diagnosis and treatment displacement rates.

## Graphical abstract

**Figure:**
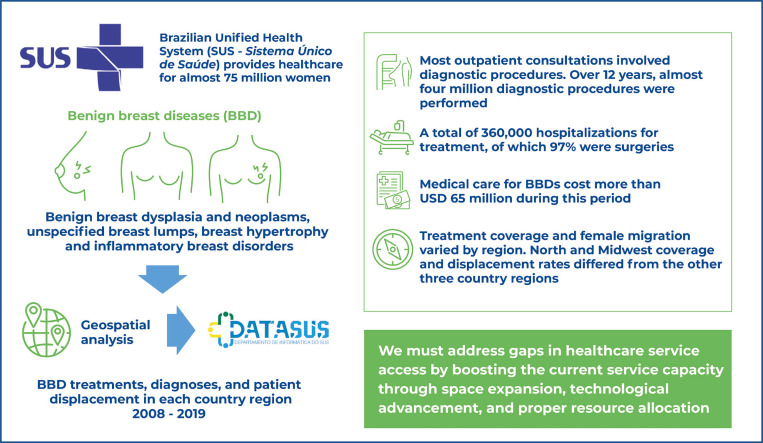

## INTRODUCTION

Universal healthcare systems, such as the Brazilian Unified Health System (SUS - *Sistema Único de Saúde*), face ongoing issues in countries of continental proportions and significant social and geographical inequities. Decentralization is a critical component in the implementation of SUS logistics. Primary care, supported by decentralization, ensures universal access to a wide range of services, including coordination for more complex treatments such as medical hospital care. Dealing with the increasing demand amidst rapid and frequent technological developments is challenging for a robust public healthcare system such as SUS.^1^

Although population aging and lifestyle changes further affect female nonreproductive health, healthcare systems, particularly in developing countries, are ill-prepared to cope with this “new” concern.^2,3^ This concern is particularly relevant for Brazil because according to the Brazilian Institute of Geography and Statistics (IBGE - *Instituto Brasileiro de Geografia e Estatística*) 2010 Census, the country has 98 million women^4^ and only 25% receive any form of supplementary healthcare, leaving nearly 75 million women reliant solely on the SUS.^5^

Benign breast diseases (BBDs) encompass several diagnoses, including fibroadenomas, cysts, fibrocystic disorders, papillomas, and ductal epithelial proliferation.^6-8^ The conditions deemed as BBDs by the International Classification of Diseases (ICD)-10^9^ include benign breast dysplasia (N60), inflammatory breast disorders (N61), breast hypertrophy (N62), unspecified breast lumps (N63), other breast diseases (N64), benign neoplasms (D24), and breast neoplasms with uncertain or unknown behavior (D48.6).

Benign breast diseases are common and can potentially increase the risk of developing breast cancer.^6,10^ Some studies have reported a 4- to 5-fold higher risk in patients with BBDs and atypia and a 1.5-2-fold greater risk in those without atypia.^11^ However, determining the prevalence and incidence of BBDs is challenging because these conditions receive little clinical attention,^12^ and the lack of a standardized histological classification system hinders diagnosis.^6^ The cumulative incidence of biopsy-proven BBDs ranges from 10 to 20%.^13^

Spatial epidemiology, which combines geography and public health, uses geospatial analytical tools to answer disease-related questions. It provides a valuable tool for strategic planning in public administration^14^ by contextualizing health events, aiding the understanding of the socio-environmental processes involved in their occurrence, and assisting in overcoming the significant challenge of adapting service demand to supply. Therefore, this study was a quantitative, descriptive, and exploratory analysis of BBD care in the SUS from 2008 to 2019. We considered the number, cost, and spatial distribution of procedures, hospitalizations, and patient migration for BBD diagnosis and treatment.

## OBJECTIVE

This study aimed to analyze the management of benign breast diseases within the Brazilian Unified Health System, focusing on the number and geospatial distribution of procedures, costs, hospitalizations, and patient migration for benign breast disease diagnosis and treatment.

## METHODS

All evaluated data are available in the Department of Informatics of the Brazilian Unified Health System (DATASUS - *Departamento de Informação e Informática do Sistema Único de Saúde*) databases.^15^ The Hospital Information System (SIH - *Sistema de Informações Hospitalares*) supplied hospital data, and the Outpatient Information System (SIA) provided outpatient data. DATASUS data were downloaded in. dbf format using the file transfer protocol via FileZilla software and then connected to DATASUS at ftp://ftp.datasus.gov.br. Institutional Review Board approval was not required as this study used publicly available secondary data.

The study population included all women registered in these databases from 2008 to 2019 with any BBD described in the ICD-10, including benign breast dysplasia (N60), inflammatory breast disorders (N61), breast hypertrophy (N62), unspecified breast lumps (N63), other breast diseases (N64), benign neoplasms (D24), and breast neoplasms with uncertain or unknown behavior (D48.6). The expense values for outpatient procedures and admissions are presented in US dollars (USD). The conversion rate was calculated as the mean annual exchange rate (Table 1S, Supplementary Material).^16^

**Figure 1:**
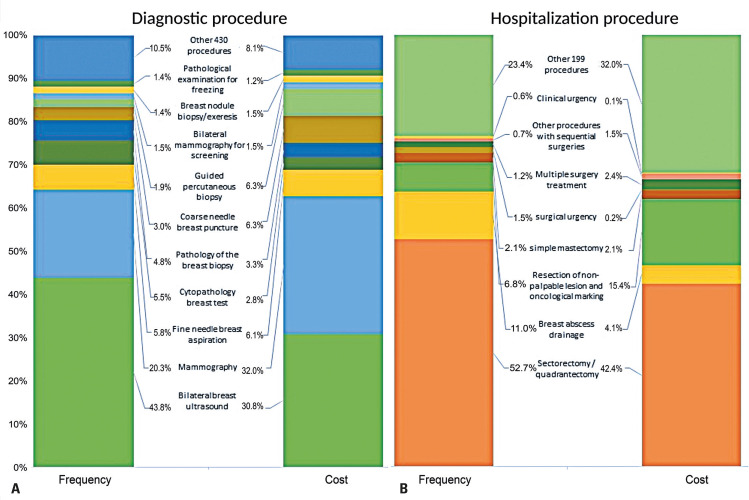
Diagnostic procedure and hospitalization frequency and costs. A) Diagnostic procedure; B) hospitalization

We constructed new tables from the DATASUS database using a Python application specifically designed to extract data from the .dbf files of DATASUS. We used IBGE mesoregions to create a spatial cutout grouping of multiple municipalities and divided Brazil into 137 territories^17^ (Figure 1S, Supplementary Material).

We used R software (R Foundation for Statistical Computing, Vienna, Austria) to construct the displacement and migration graphics. Distances between municipalities were calculated using their centroids, and graphics were created using the ‘circlize’ package. All other calculations and analyses were performed in Microsoft Excel (Microsoft Corporation, Redmond, WA, USA). The *t*-test and Kruskal-Wallis and Dunn’s *post-hoc* tests were used to analyze data. A p-value of 5% was used to determine differences in treatment coverage and migration between Brazilian regions.^18^ The QGIS georeferencing tool was used to create maps, and population data were obtained from the IBGE 2010 census.^4^

## RESULTS

Over 12 years of outpatient and inpatient care in the SUS, all six BBD-related ICD records were continuous. On average, 362,000 treatments were conducted annually in the outpatient setting, including 94.96% diagnostic procedures costing USD 5.42 million. Between 2008 and 2019, 4,349,877 BBD-related procedures were performed at a total cost of USD 65,029,686.11 (Table 2S, Supplementary Material).

The most commonly performed procedure was bilateral breast ultrasonography, with an average of 158,926.4 procedures annually at a cost of USD 1,669,811.58. Mammography was the second most common procedure, with an annual average of 73,755.80 procedures and an average yearly cost of USD 1,732,184.75. Notably, mammograms were conducted more often throughout the first 2 years of the study (569,019 in 2008 and 313,439 in 2009) (Figure 1A and Table 3S, Supplementary Material). A total of 138,038 clinical and surgical procedures were undertaken, accounting for 3.2% of the total costs. These procedures included dressing, drainage, sedation, and consultations.

Over 12 years of inpatient care, 363,112 hospitalizations were conducted at a total cost of USD 88,999,026.43 (Table 4S, Supplementary Material). Patients stayed in the hospital for an average of 1.69 days. Intensive care unit (ICU) admissions were necessary in only 847 hospitalizations, with an average stay of 2.93 days per admission. Most hospitalizations (96.78%) were surgical, mainly sectorectomies or quadrantectomies. More than 191,000 hospitalizations, costing USD 37 million, were conducted over the 12 years (Figure 1B and Table 5S, Supplementary Material). The second most common procedure was breast abscess drainage, which was performed almost 40,000 times over the 12 years.

Benign breast diseases accounted for 0.26% of all hospitalizations in the SUS over the 12 years, corresponding to 0.15% of all financial resources expended. These admissions cost only 0.09% of the total hospital ward bed payments and 0.004% of the ICU bed payments per day. BBDs used 0.08% of the resources for outpatient procedures, accounting for 0.01% of all procedures performed under the SUS (Table 6S, Supplementary Material).

**Figure 2:**
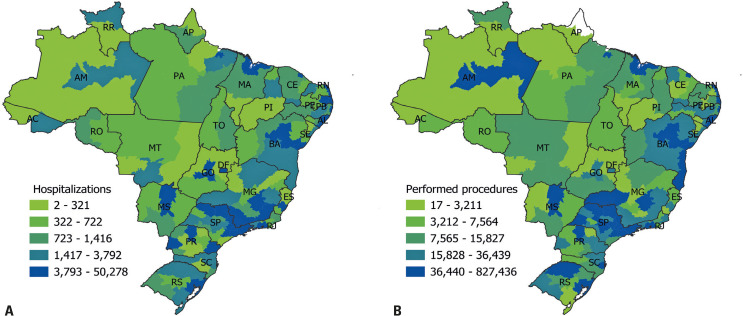
Patient care for benign breast diseases per Brazilian mesoregion. A) Inpatient care; B) outpatient care

**Figure 3:**
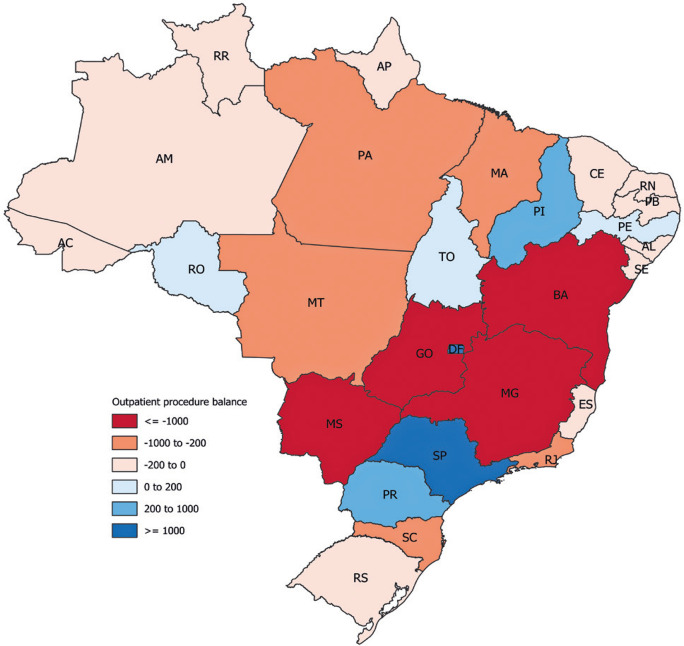
Balance of inflow and outflow of patient migration for outpatient procedures

The mean ages of outpatients (46.86 years) (Figure 2S, Supplementary Material) and hospitalized patients (38.57 years; Figure 3S, Supplementary Material) significantly differed (*t*-test, p<0.000).

### Spatial distribution: inpatient care

Hospitalizations were concentrated in a few mesoregions, with 51.73% occurring in 13 of 137 Brazilian mesoregions. Hospitalizations for BBD were more common in the metropolitan regions of São Paulo (50,278; 13.65%), Rio de Janeiro (18,723; 5.08%), Belo Horizonte (14,603; 4.02%), Fortaleza (14,024; 3.81%), and Recife (13,253; 3.60%) (Figure 2A). Hospitalization coverage (*i.e*., the number of hospitalizations per 100,000 inhabitants) significantly differed among the five major Brazilian regions (Kruskal-Wallis, p=0.0495) (Table 7S, Supplementary Material). The Northern region had fewer hospitalizations (105.9 per 100,000 people) than those of the Southeast region (158.7 per 100,000 inhabitants) (Dunn’s test, p=0.02). The Southeast region had higher hospitalizations than did the Midwest region (113.2 per 100,000 inhabitants) (Dunn’s test, p=0.03). However, the Northeast (155.1 per 100,000 inhabitants) and South (152.9 per 100,000 inhabitants) showed no significant differences from the other regions (Dunn’s test, p=0.08, p=0.47, p=0.88, p=0.08 and p=0.09, p=0.88, p=0.64, and p=0.09, respectively) (Table 8S, Supplementary Material).

### Spatial distribution: outpatient care

Only 9 of the 137 mesoregions accounted for 50.15% of outpatient care. The metropolitan areas of São Paulo (827,436; 19.02%), Salvador (395,682; 9.10%), Rio de Janeiro (202,536; 4.66%), Vale do Paraíba Paulista (161,253; 3.71%), and Central North Bahia (153,541; 3.53%) had the highest number of visits (Figure 2B).

Outpatient care coverage, *i.e*., the number of outpatient procedures per 100,000 inhabitants, differed across the five major Brazilian regions (Kruskal-Wallis test; p=0.0060) (Table 9S, Supplementary Material). Procedural coverage in the North region (924.8 per 100,000 inhabitants) was much lower than in the Northeast (1,737.7 per 100,000 inhabitants) (Dunn’s test, p=0.01), Southeast (1,947.2 per 100,000 inhabitants) (Dunn’s test, p<0.00), and South (1,679.6 per 100,000 inhabitants) (Dunn’s test, p=0.01) regions. Procedural coverage in the Midwest (1,039.6 per 100,000 inhabitants) was lower than that in the Southeast region (Dunn’s test, p=0.02) (Table 10S, Supplementary Material).

### Migrations: inpatient care

Several patients travel to other cities for BBD treatment, and some treatments require patients to travel between cities, mesoregions, federation units, or even regions.

**Figure 4:**
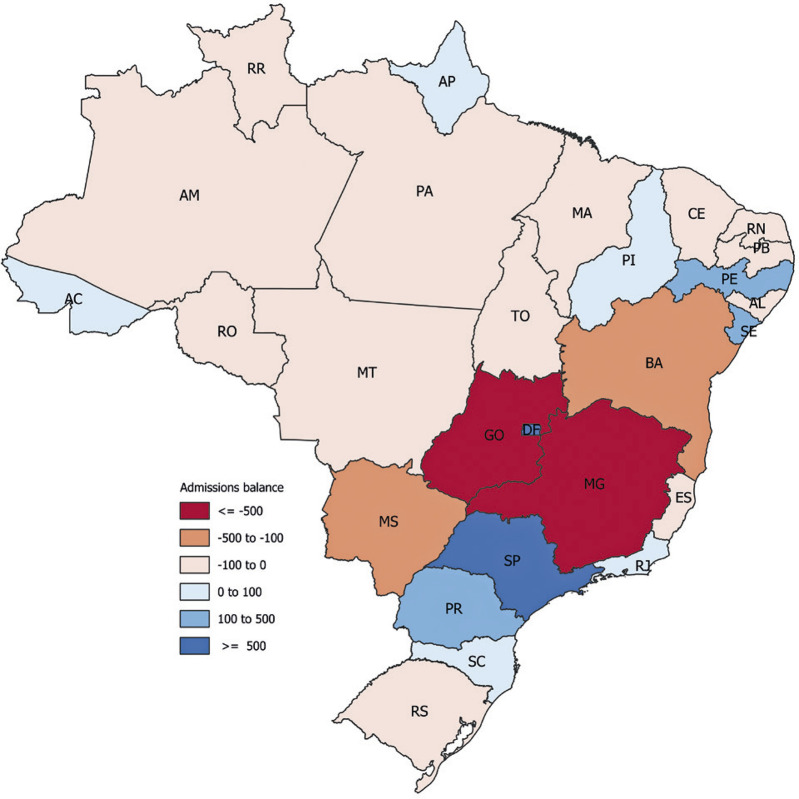
Balance of inflow and outflow of patient migration for hospitalization

Patients traveled to another mesoregion for 10.72% of hospitalizations. Depending on the patient’s origin, the migration rate ranged from 6.53 to 16.21% (Table 11S, Supplementary Material). The greatest distance traveled for admission was in the Northern region. Hospitalization displacement requirements differed across the five major Brazilian regions (Figure 4S, Supplementary Material) albeit not significantly (Kruskal-Wallis test, p=0.331) (Figure 5S, Supplementary Material).

Similar to outpatient care, hospitalizations showed disparities in the origin and destination of migration. The South and Southeast regions received more people for hospital admissions than they sent, and the North, Northeast, and Midwest regions sent more patients than they received (Figure 3). Figures 6S-10S in the Supplementary Material show the migration graphs for all services conducted beyond the original mesoregion of the patient.

### Migrations: outpatient care

Considering the total number of procedures, 5.92% of the patients required travel to different mesoregions. The migration rates varied considerably, with the Southeast having the lowest rate at 2.94% and the Midwest having the highest at 14.58% (Table 12S, Supplementary Material). Travel requirements varied across the country (Figure 11S, Supplementary Material), but with no significant differences (Kruskal-Wallis, p=0.172) (Figure 12S, Supplementary Material).

Although migration between regions did not differ, the origins and destinations of migration differed. The North, Northeast, and Midwest regions showed a negative balance, as more patients left their mesoregions for procedures than those arriving. Conversely, the South and Southeast regions experienced a positive balance, with more patients entering for treatment than leaving. The red areas in figure 4 indicate a high outflow of patients, whereas the blue regions show an increased inflow. Figures 13S-17S, Supplementary Material, depict migration for outpatient care between mesoregions.

### Migrations: distance traveled

Analysis of the distances traveled by patients for outpatient procedures and hospitalization outside their home regions showed that the distance varied significantly. The average distance was 314.4 km for hospitalizations and 378.8 km for outpatient visits. The North and Midwest regions had the highest mean distances traveled for hospitalization and outpatient procedures. The distances traveled for in- and outpatient procedures significantly differed between regions (Kruskal-Wallis, p<0.000 and 0.002, respectively) (Figure 18S and Table 13S, Supplementary Material).

Regarding the distance to reach hospitals, the North (mean, 774.7 km) and Midwest (mean, 388.0 km) regions did not differ significantly (Dunn’s test, p=0.48). However, these two regions accounted for the most distance than the other three regions (Northeast, mean, 226.9 km; Southeast, mean, 181.8 km; South, mean, 239.1 km) (Dunn’s showed p<0.00 for the three regions compared with the Northern region and p<0.01 compared with the central-west region) (Figure 19S, Supplementary Material).

Concerning outpatient care, the North (mean, 823.8 km) and Midwest (mean, 434.2 km) regions were similar in terms of distance traveled (Dunn’s test, p=0.96). Nevertheless, their distances were significantly higher than those of the Southeast (mean, 240.6 km) (Dunn’s test, p<0.0021 for North and Midwest) and South (mean, 213.6 km) (Dunn’s test, p<0.0021 for North and Midwest) regions. The Northeast region (mean, 350.3 km) did not significantly differ from any other region (Dunn’s test, p=0.12 *versus* North, p=0.055 *versus* Midwest, p=0.06 *versus* Southeast, p=0.18 *versus* South). The distances traveled in the Southeast and South regions were similar (Dunn’s test p=0.88) (Table 14S, Supplementary Material).

## DISCUSSION

Understanding BBDs is critical for planning prevention strategies, alleviating associated symptoms, and ruling out malignant neoplasms with similar clinical presentations. Consequently, outlining BBD prevention strategies and simultaneously alleviating associated symptoms is possible.

Our results demonstrated that most patients in outpatient clinics underwent diagnostic procedures, as more than 95% of the procedures were diagnostic. An approximately 60% decline in the number of procedures was observed from 2008 to 2010, 2 years after the start of our analysis. This decrease resulted from the reduction in the number of mammograms. After 2009, this procedure was no longer considered in the SUS databases for BBD diagnosis, reappearing only in 2015 and in minimal numbers.

Notably, drug dispensing is among the therapeutic measures listed in the SIA databases, accounting for a significant proportion of outpatient therapeutic procedures for most diseases. Outpatient treatment and clinical and surgical procedures accounted for only 3.2% of all the procedures performed (138,000 in 12 years). This phenomenon is unusual in the SUS. However, most medications used to treat BBDs, such as danazole, bromocriptine, tamoxifen, vitamin E, medroxyprogesterone acetate, progesterone, and gonadotropin-releasing hormone,^19-25^ are not registered for this use in Brazil or have not yet been listed among the drugs supplied by the SUS for BBDs. In both cases, the outcome is the same; the SUS did not provide medication for BBD treatment. Our analysis revealed that outpatient procedures for diagnostic purposes were overrepresented.

In contrast, the BBD treatments reported by DATASUS were primarily surgical, *e.g.*, sectorectomies and quadrantectomies, and involved hospitalization. Surgeries were conducted at a similar frequency across the 12 years. When it is unknown whether a patient has a malignant or benign tumor, surgery to remove a non-palpable breast lesion marked using breast oncological techniques (the third most common and second most expensive procedure in our analysis) is frequently performed in a hospital setting to rule out the possibility of breast cancer. SIH includes this technique among those permitted for patients with BBDs listed in the ICD-10. As a result, some hospitalizations may have occurred for diagnosis rather than treatment of a previously diagnosed BBD.

The age of women treated for BBD in the SUS was consistent with that reported in literature.^26^ The in- and outpatient age distributions showed two peaks: one at 19 years and the other at 45 years. The initial peak in the age distribution graph of outpatients is more subtle. Changes in the anatomical and histological structures of the breast throughout the initial reproductive years and at the start of menopause may explain these peaks.^27,28^ We hypothesize that the therapeutic procedures for BBD treatment were conducted in younger women. In contrast, the procedures performed on older females in the second peak aimed to rule out potential malignant tumors.

The spatial distribution of BBD treatments and diagnoses in the Brazilian territory is uneven. Brazil’s three most populated regions (Southeast, Northeast, and South) had a higher average number of hospitalizations per inhabitant, with approximately 150 hospitalizations per 100,000 inhabitants. In contrast, the Midwest and North regions had approximately 110 hospitalizations per 100,000 inhabitants. This discrepancy was also observed for outpatient procedures, as the former regions had over 1,800 procedures per 100,000 residents, while the latter had only approximately 1,000 procedures per 100,000 residents. This difference in coverage, which is 30-40% lower in the North and Midwest regions, may be due to the disparity in healthcare services and patient migration from these regions.

The lower number of diagnostic procedures performed in more depopulated areas suggests the possibility that BBDs are underdiagnosed. This underdiagnosis would result in even lower treatment coverage than in other regions because many patients still require a diagnosis. Inequity in services across territories forces patients to migrate from one area to another.

Several hospitalizations and outpatient procedures occur far from the patient’s homes due to the lack of comprehensive clinical-medical infrastructure in each city. This arrangement is expected and planned in an integrated healthcare system such as the SUS, especially in a vast country such as Brazil. Patients often have to leave their towns and mesoregions for diagnosis and treatment, sometimes traveling to another federal unit or region to receive medical care. However, migration from a mesoregion for BBD diagnosis, which is not a high-complexity procedure, is uncommon.

Patients from other mesoregions accounted for 6.15% of all procedures performed, requiring an average travel distance of 378 km for these basic procedures. Less populated areas have a higher requirement for patient displacement, revealing spatial variability in healthcare provision. The Midwest region had the highest patient migration rate, with 14.58% of procedures performed in a mesoregion other than that of the patient’s origin. It is important to note that intra-mesoregion mobility was not considered in this analysis.

The need to migrate for hospitalization was consistent with outpatient treatment, albeit at a slightly higher rate (>10%). This need is not surprising, given that the diagnostic complexity of BBD (ultrasound, mammography, and punctures) is lower than the treatment complexity, which is typically surgical in Brazil.

Although Brazil has mechanisms to deal with population displacement in these situations,^29^ distances greater than 1,000 km are typical in the North and Midwest regions. The need for long journeys to diagnose BBD potentially reveals an imperfection in the healthcare system. Women from poorer and more depopulated areas have to travel long distances because they lack sufficient healthcare infrastructure in their mesoregions for low-complexity procedures.

The SUS expenditure for BBD diagnosis and treatment was minimal. BBD-related expenditures have negligible impact on a healthcare system with a structure as substantial as the SUS, with millions of dollars spent annually. In the 12 years, BBDs represented less than one-thousandth of the money spent on SUS outpatient clinics and hospitalizations. This low representation most likely occurs because after diagnosing a non-malignant breast disease, treatment usually aims to control and treat symptoms and educate patients. As previously stated, the SUS does not provide drugs for the treatment of BBD. Therefore, although BBDs require fewer systemic resources, access to adequate therapies is limited.

Differentiating BBDs from malignant neoplasms is critical for both patients and healthcare systems. Because of this distinction, Brazilian databases have revealed that surgeries, such as sectorectomies and quadrantectomies, are more prevalent, whereas access to pharmacological therapies for BBDs is limited. A public and universal healthcare system, such as the SUS, expects patients to migrate for diagnosis or treatment. This expectation has generated procedures for dealing with patient migration in these instances. When these displacements for low- and medium-complexity procedures such as BBD diagnosis and treatment occur, the system needs improvement such that women from less economically favored and more depopulated areas do not travel long distances to receive medical assistance.

The strategies for system improvement are undeniably complex. However, some elements deserve consideration, such as preventing and treating non-communicable diseases and ensuring access to tests and treatments for conditions such as BBDs. This effort is critical because non-communicable diseases accounted for 7 of the 10 global leading causes of death in 2019. Therefore, addressing disparities in access to healthcare services, regardless of their source, is critical. This can be achieved by expanding the primary care network to incorporate population health services, including outpatient clinics, basic health units, and emergency care.

Furthermore, the service capacities of existing facilities must be enhanced through spatial expansion and technological advancement. Similarly, the correct allocation of resources by the healthcare system must be ensured by training management professionals and capacitating frontline healthcare workers in the science, technology, and information fields. This set of actions is only a proposal to accelerate access to tests and medicines for the entire population and boost the quantity and quality of healthcare, particularly for those experiencing poverty. However, their execution is complex.

## CONCLUSION

Service deficiencies and significant disparities in coverage within populated areas underscore the need to enhance the healthcare network, encompassing outpatient clinics, basic health units, and emergency care. Additionally, augmenting the service capacity of existing facilities through spatial expansion and technological advancement is essential.
