# Supplementary material for: Brazil’s benign breast disease care profile and geospatial analysis
Source: Einstein (Sao Paulo). 2025 Mar 28;23:eAO1132. doi: 10.31744/einstein_journal/2025AO1132 (PMC12014158; doi:10.31744/einstein_journal/2025AO1132)
Supplement: Supplementary file 1 [file 2317-6385-eins-23-eAO1132-suppl01.pdf]

SUPPLEMENTARY MATERIAL

Brazil's benign breast disease care profile and geospatial analysis

Dayan Sansone, Daniela Farah, Afonso Celso Pinto Nazario, Marcelo Cunio Machado Fonseca

DOI: 10.31744/einstein_journal/2025AO1132

Table 1S. Conversion rate of reais (R$) to US dollar (US$) for each year

Year	R$	US$
2008	1.8338	1.00
2009	1.9968	1.00
2010	1.7594	1.00
2011	1.6742	1.00
2012	1.954	1.00
2013	2.1570	1.00
2014	2.3529	1.00
2015	3.3309	1.00
2016	3.4895	1.00
2017	3.1914	1.00
2018	3.6536	1.00
2019	3.9445	1.00

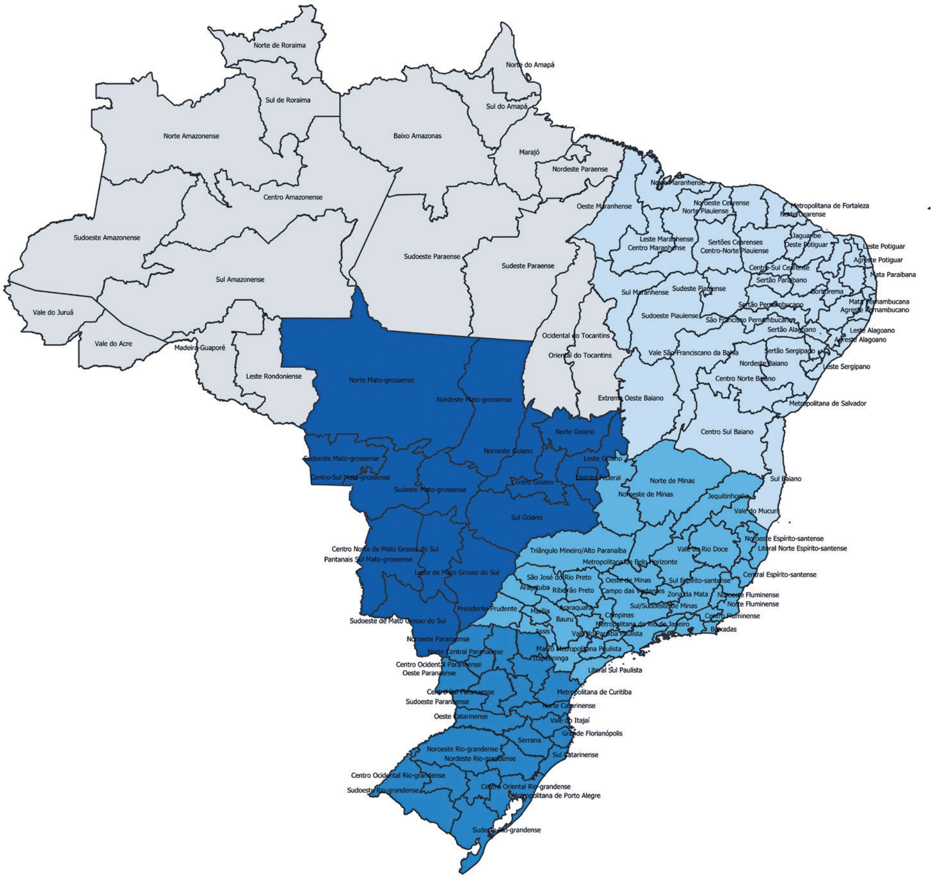

Figure 1S. Brazilian mesoregions

Table 2S. Quantity and costs of procedures performed for benign breast diseases from 2008 to 2019

Year	Number of procedures	Cost
2008	802,322	$16,958,670.82
2009	609,667	$11,112,251.64
2010	317,621	$5,083,760.26
2011	301,016	$5,282,644.62
2012	270,761	$4,100,461.05
2013	273,174	$3,688,900.30
2014	283,760	$3,555,971.44
2015	290,596	$2,655,401.62
2016	307,903	$2,709,765.57
2017	267,805	$2,713,396.18
2018	287,113	$3,388,914.39
2019	338,139	$3,779,548.22
Total	4,349,877	$65,029,686.11

Table 3S. Procedures performed for benign breast diseases from 2008 to 2019

Medical procedure	Number of procedures	Cost
Bilateral breast ultrasound	1,907,117	$20,037,738.90
Mammography	885,069	$20,786,217.06
Fine needle breast aspiration	254,134	$3,997,322.92
Cytopathology breast test	241,114	$1,806,025.89
Pathology of the breast biopsy	206,798	$2,160,831.30
Coarse needle breast puncture	129,633	$4,116,271.26
Guided percutaneous biopsy	82,791	$4,118,256.06
Bilateral mammography for screening	63,421	$988,649.49
Breast nodule biopsy/exeresis	61,799	$982,993.01
Pathological examination for freezing	60,787	$798,990.78
Other 430 procedures	457,060	$5,236,389.44

Table 4S. Hospitalizations, costs, hospital days, and hospital days in the intensive care unit for benign breast diseases from 2008 to 2019

Year	Number of hospitalizations	Hospitalizations cost	Hospital stay (days)	ICU stay (days)
2008	25,420	$4,887,265.92	44,165	66
2009	30,067	$5,905,011.30	50,043	122
2010	30,673	$7,025,856.19	51,474	211
2011	30,045	$7,450,050.73	51,545	88
2012	30,278	$6,836,233.80	51,529	188
2013	30,823	$10,825,103.27	53,504	149
2014	33,558	$12,381,807.51	56,524	232
2015	30,424	$6,928,507.96	52,729	264
2016	29,847	$6,424,589.64	51,657	250
2017	29,930	$7,413,236.31	51,914	264
2018	29,963	$6,395,640.77	54,165	327
2019	32,084	$6,525,723.04	56,588	319
Total	363,112	$88,999,026.43	625,837	2,480

ICU: intensive care unit.

Table 5S. Procedures performed during hospitalization for benign breast diseases from 2008 to 2019

Procedures	Number	Costs
Sectorectomy/quadrantectomy	191,312	$37,719,506.20
Breast abscess drainage	39,893	$3,683,968.37
Resection of non-palpable lesions and oncological marking	24,831	$13,736,555.07
Simple mastectomy	7,660	$1,826,036.10
Surgical urgency	5,332	$143,006.15
Multiple surgery treatment	4,387	$2,094,096.09
Other procedures with sequential surgeries	2,447	$1,292,340.80
Clinical urgency	2,223	$54,162.38
Other 199 procedures	85,027	$28,449,355.27

Table 6S. Out- and inpatient procedures and costs for benign breast diseases compared with total Brazilian Unified Health System costs from 2008 to 2019

	Total SUS	Total BBD	%
Outpatient			
Procedures performed	44,167,290,610	4,349,723	0.010
Cost of procedures	$81,441,940,667.15	$65,029,686.11	0.080
Inpatient			
Hospitalizations	139,630,238	363,112	0.260
Hospitalizations cost	$60,806,973,981.77	$88,999,026.43	0.146
ICU cost	$11,352,470,136.36	$432,586.79	0.004
Ward bed daily rates	705,489,619	625,837	0.089
ICU bed daily rates	55,733,662	2,480	0.004

ICU: intensive care unit; BBD: benign breast diseases; SUS: *Sistema Único de Saúde*.

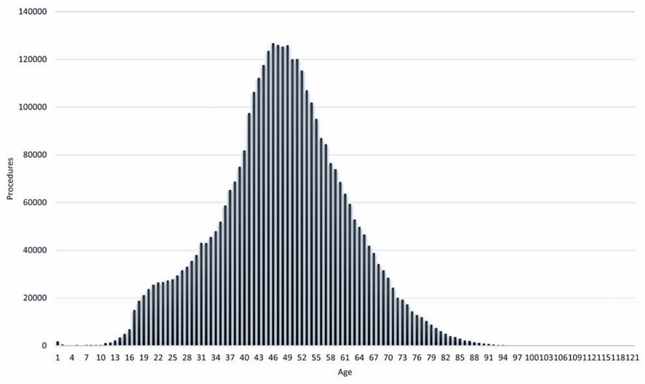

Figure 2S. Number of outpatient procedures performed for benign breast disease by patient age from 2008 to 2019

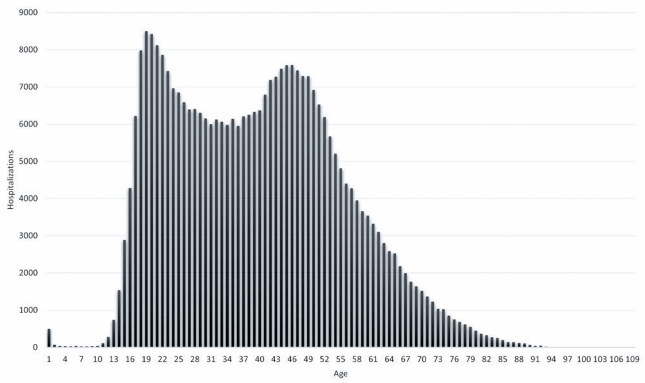

Figure 3S. Number of hospitalizations performed for benign breast disease by patient age from 2008 to 2019

Table 7S. Average hospitalizations per 100,000 inhabitants per benign breast disease in Brazilian regions from 2008 to 2019

Hospitalizations per 100,000 inhabitants	North	Northeast	Southeast	South	Midwest
Number of mesoregions	20	42	37	23	15
Minimum of hospitalizations	7.4	0.8	25.4	34.5	9.3
Maximum of hospitalizations	381.4	582.2	467.5	562.3	379.3
Average hospitalizations	105.9	155.1	158.7	152.9	113.2
Standard deviation	96.0	118.3	97.4	113.8	123.8

Table 8S. Dunn's test for differences in hospital coverage in the five major Brazilian regions from 2008 to 2019

	North	Northeast	Southeast	South	Midwest
North		0.08	0.02*	0.09	0.88
Northeast	0.08		0.47	0.88	0.08
Southeast	0.02*	0.47		0.64	0.03*
South	0.09	0.88	0.64		0.09
Midwest	0.88	0.08	0.03*	0.09	

* Significant differences between regions.

Table 9S. Average number of outpatient procedures performed per 100,000 inhabitants for benign breast disease in Brazilian regions from 2008 to 2019

Procedures per 100,000 inhabitants	North	Northeast	Southeast	South	Midwest
Number of mesoregions	20	42	37	23	15
Minimum of procedures	0.0	206.0	202.0	335.4	171.5
Maximum of procedures	3,251.0	8,721.2	6,741.3	5,730.5	3,595.5
Average of procedures	924.8	1,737.7	1,947.2	1,679.6	1,039.6
Standard deviation	903.6	1,644.3	1,544.9	1,299.7	888.2

Table 10S. Dunn's test for differences in outpatient procedure coverage in the five major Brazilian regions from 2008 to 2019

	North	Northeast	Southeast	South	Midwest
North		0.01*	0.00*	0.01*	0.58
Northeast	0.01*		0.43	0.90	0.07
Southeast	0.00*	0.43		0.58	0.02*
South	0.01*	0.90	0.58		0.09
Midwest	0.58	0.07	0.02*	0.09	

* Significant differences between regions.

Table 11S. Hospitalizations of patients with benign breast diseases outside their mesoregion of origin from 2008 to 2019

Origin of patients	Number of hospitalizations	Number of hospitalizations outside patient mesoregion	% of hospitalizations outside patient mesoregion
North	20,674	2,010	9.72
Northeast	106,356	16,557	15.57
Southeast	160,201	10,453	6.53
South	53,609	6,021	11.23
Midwest	27,390	4,440	16.21
Brazil	368,230	39,481	10.72

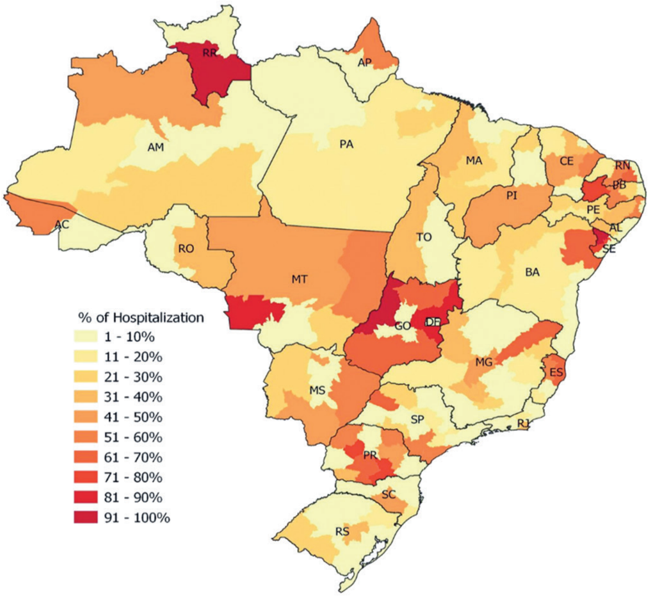

Figure 4S. Percentage of hospitalizations for benign breast diseases outside the patient's mesoregion from 2008 to 2019

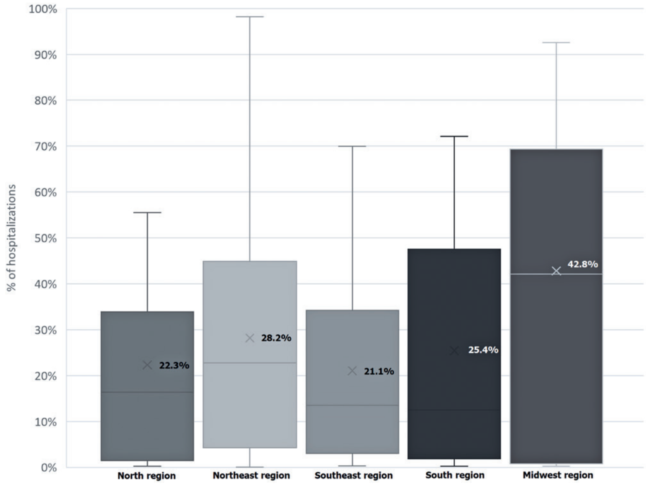

Figure 5S. Boxplot of the migration percentages out of the patient's mesoregion of origin for hospitalizations for benign breast diseases by Brazilian region from 2008 to 2019

* Figures 6S-10S show the migration graphs between the mesoregions for hospitalizations for benign breast diseases from 2008 to 2019 (the graphs are on a log2 scale for better visualization).

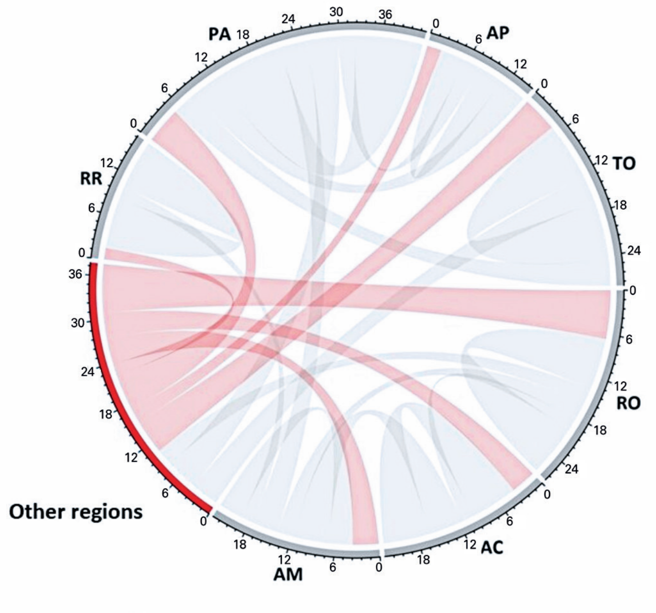

Figure 6S. Displacements from the North region (red: hospitalizations in other regions for patients coming from the North region; gray: migrations within the North region)

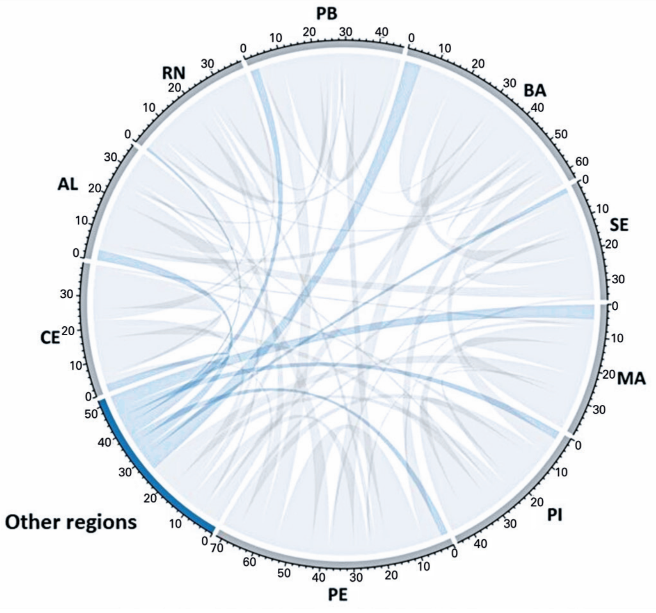

Northeast region states: CE-Ceará, AL-Alagoas, RN-Rio Grande do Norte, PB-Paraíba, BA-Bahia, SE-Sergipe, MA-Maranhão, PI-Piauí, PE-Pernambuco.

Figure 7S. Displacements from the Northeast region (blue: hospitalizations in other regions for patients coming from the Northeast region; gray: migrations within the Northeast region)

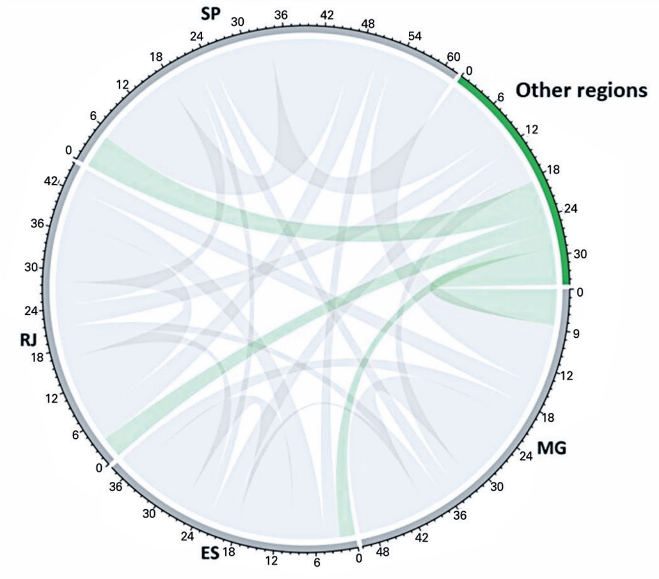

Southeast region states: MG-Minas Gerais, ES-Espírito Santo, RJ-Rio de Janeiro. SP-São Paulo.

Figure 8S. Displacements from the Southeast region (green: hospitalizations in other regions for patients coming from the Southeast region; gray: migrations within the Southeast region)

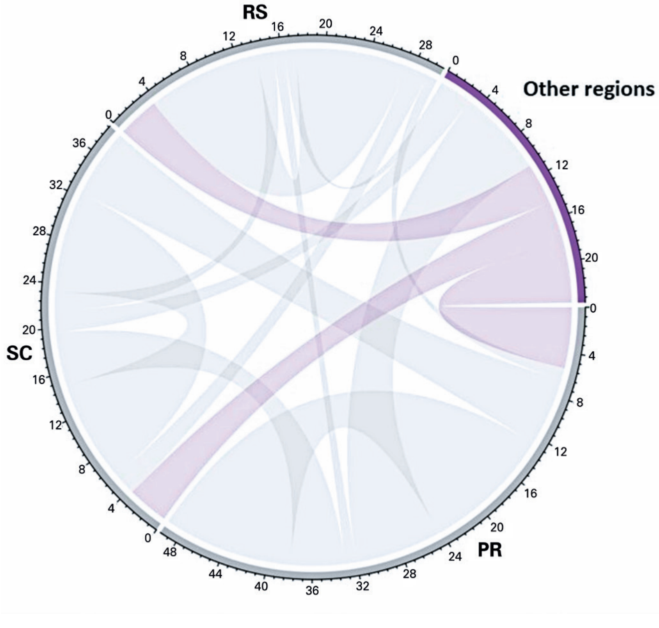

South region states: PR Paraná, SC Santa Catarina, and RS Rio Grande do Sul.

Figure 9S. Displacements from the South region (purple: hospitalizations in other regions for patients coming from the South region; gray: migrations within the South region)

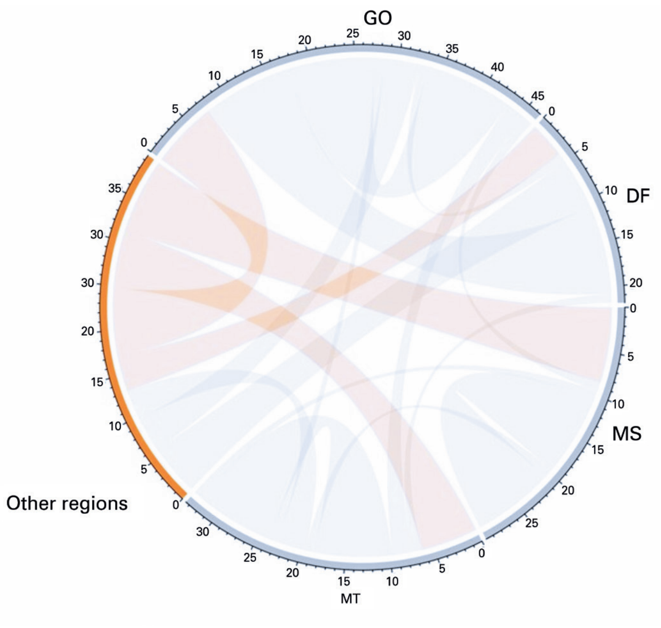

Midwest states: GO Goiás, DF Distrito Federal, MS Mata Grosso do Sul, and MT Mato Grosso.

Figure 10S. Displacements from the Midwest region (orange: hospitalizations in other regions for patients coming from the Midwest region; gray: migrations within the Midwest region)

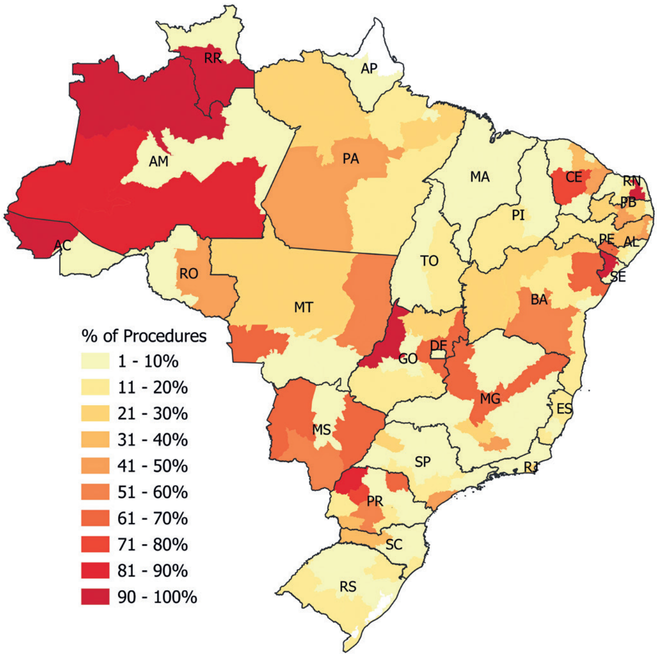

Figure 11S. Percentage of outpatient procedures for benign breast diseases outside the patient's mesoregion of origin from 2008 to 2019

Table 12S. Outpatient procedures performed for patients with benign breast disease outside their mesoregion of origin from 2008 to 2019

Origin of patients	Procedures performed	Procedures performed outside mesoregion of origin	% of procedures for which patients changed mesoregion
North	212,356	15,223	7.17
Northeast	1,402,264	124,610	8.88
Southeast	2,066,135	60,657	2.94
South	501,452	32,564	6.49
Midwest	167,156	24,377	14.58
Brazil	4,349,723	257,432	5.92

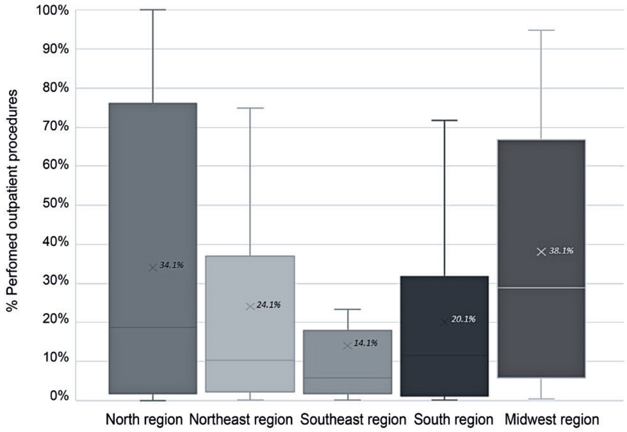

Figure 12S. Box plot of the migration percentages out of the patient's mesoregion of origin for outpatient procedures for benign breast diseases by Brazilian region from 2008 to 2019

* Figures 13S-17S show the migration graphs between mesoregions for outpatient procedures for benign breast diseases from 2008 to 2019 (the graphs are on a log2 scale for better visualization).

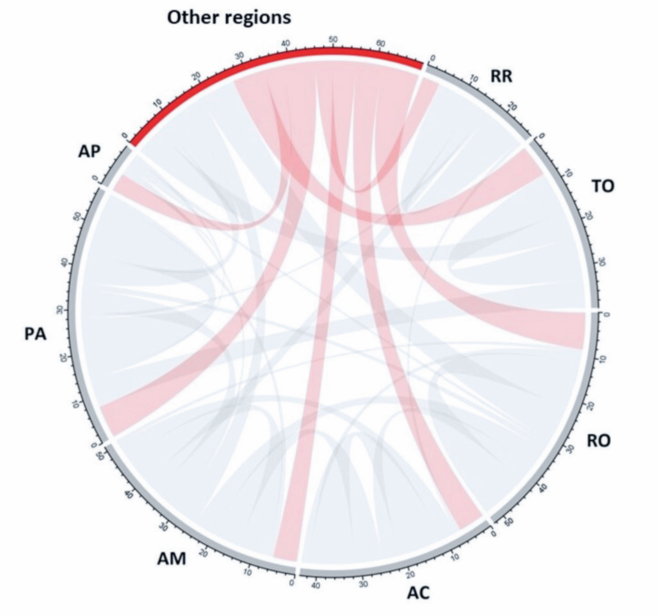

North region states: RR-Roraima, TO-Tocantins, RO-Rondônia, AC-Acre, AM-Amazonas, PA-Pará, AP-Amapá.

Figure 13S. Displacements from the North region (red: outpatient procedures in other regions for patients coming from the North region; gray: migrations within the North region)

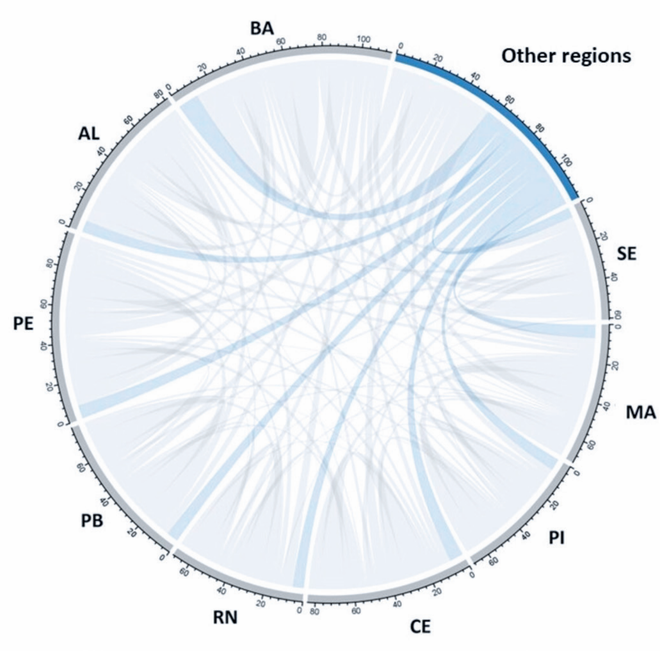

Northeast region states: SE-Sergipe, MA-Maranhão, PI-Piauí, CE-Ceará, RN-Rio Grande do Norte, PB-Paraíba, PE-Pernambuco, AL-Alagoas, BA-Bahia.

Figure 14S. Displacements from the Northeast region (blue: outpatient procedures in other regions for patients coming from the Northeast region; gray: migrations within the Northeast region)

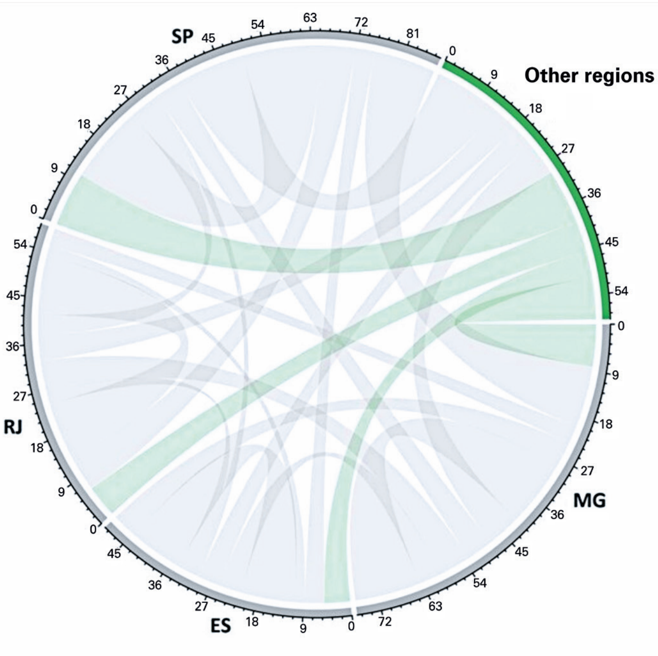

Southeast region states: MG-Minas Gerais, ES-Espírito Santo, RJ-Rio de Janeiro. SP-São Paulo.

Figure 15S. Displacements from the Southeast region (green: outpatient procedures in other regions for patients coming from the Southeast region; gray: migrations within the Southeast region)

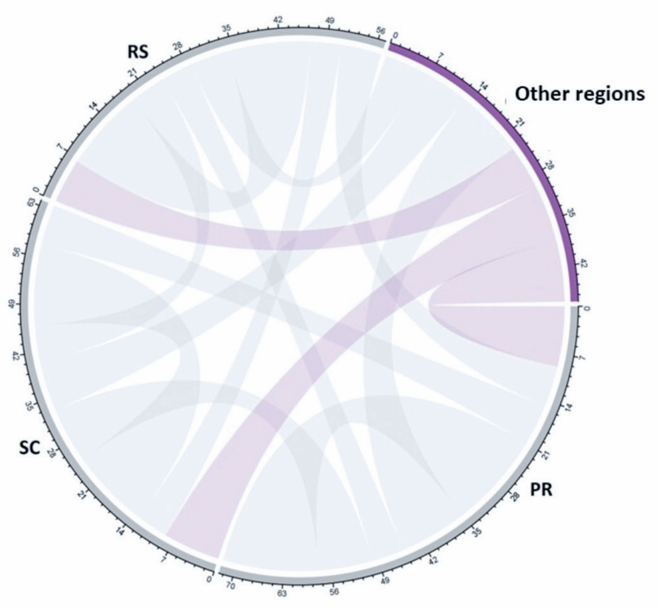

South region states: PR Paraná, SC Santa Catarina, and RS Rio Grande do Sul.

Figure 16S. Displacements from the South region (purple: outpatient procedures in other regions for patients coming from the South region; gray: migrations within the South region)

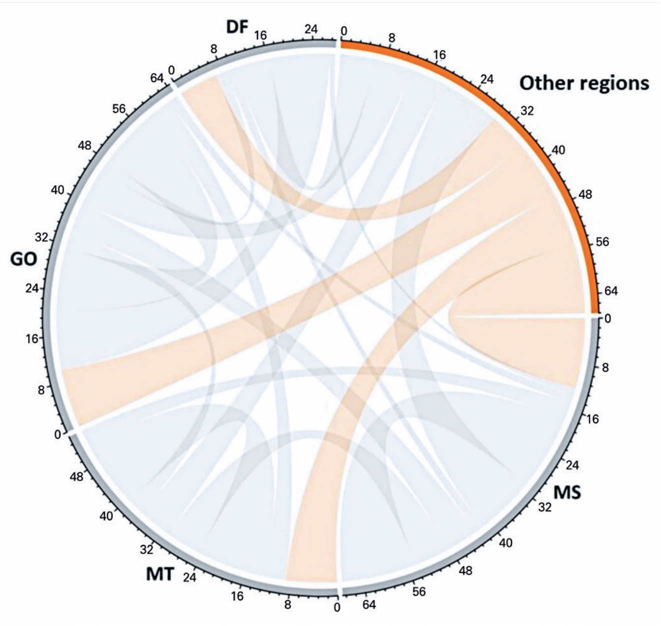

Midwest region states: MS-Mata Grosso do Sul, MT-Mato Grosso, GO-Goiás, and DF-Distrito Federal.

Figure 17S. Displacements from the Midwest region (orange: outpatient procedures in other regions for patients coming from the Midwest region; gray: migrations within the Midwest region)

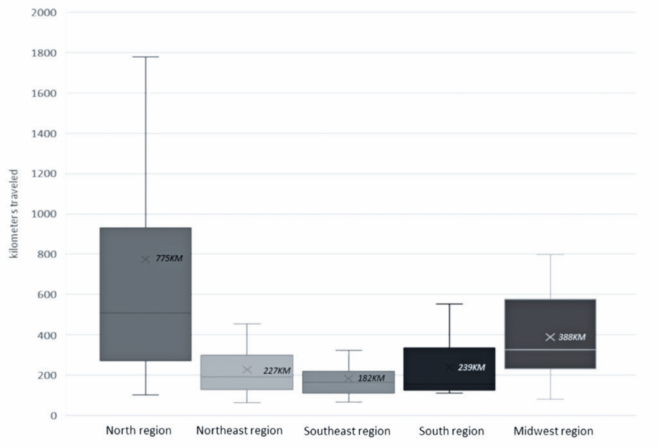

Figure 18S. Box plot of the distances covered by patients who migrated from their mesoregion of origin for hospitalization for benign breast diseases by Brazilian region from 2008 to 2019

Table 13S. Dunn's test for differences in distances covered for hospitalization in the five major Brazilian regions from 2008 to 2019

	North	Northeast	Southeast	South	Midwest
North		0.0000*	0.0000*	0.0004*	0.4835
Northeast	0.0000*		0.2349	0.9013	0.0038*
Southeast	0.0000*	0.2349		0.2586	0.0002*
South	0.0004*	0.9013	0.2586		0.0115*
Midwest	0.4835	0.0038*	0.0002*	0.0115*	

* Significant differences between regions.

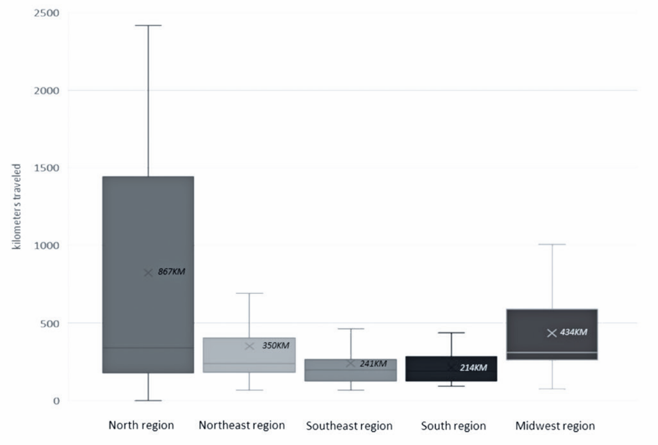

Figure 19S. Box plot of the distances covered by patients who migrated from their mesoregion of origin for outpatient procedures for benign breast diseases by Brazilian region from 2008 to 2019

Table 14S. Dunn's test for differences in distances covered for outpatient procedures in the five major Brazilian regions from 2008 to 2019

	North	Northeast	Southeast	South	Midwest
North		0.1256	0.002243*	0.003713*	0.9691
Northeast	0.1256		0.05535	0.06942	0.1804
Southeast	0.002243*	0.05535		0.8833	0.006377*
South	0.003713*	0.06942	0.8833		0.008459*
Midwest	0.9691	0.1804	0.006377*	0.008459*	

* Significant differences between regions.
